# Fluorescent Probes for Selective Recognition of Hypobromous Acid: Achievements and Future Perspectives

**DOI:** 10.3390/molecules26020363

**Published:** 2021-01-12

**Authors:** Yuyu Fang, Wim Dehaen

**Affiliations:** 1 State Key Laboratory of Southwestern Chinese Medicine Resources, School of Pharmacy, Chengdu University of Traditional Chinese Medicine, Chengdu 611137, China; yyfang@cdutcm.edu.cn; 2 Department of Chemistry, KU Leuven, Celestijnenlaan 200f-bus 02404, 3001 Leuven, Belgium

**Keywords:** fluorescent probes, selective recognition, ROS, hypobromous acid

## Abstract

Reactive oxygen species (ROS) have been implicated in numerous pathological processes and their homeostasis facilitates the dynamic balance of intracellular redox states. Among ROS, hypobromous acid (HOBr) has a high similarity to hypochlorous acid (HOCl) in both chemical and physical properties, whereas it has received relatively little attention. Meanwhile, selective recognition of endogenous HOBr suffers great challenges due to the fact that the concentration of this molecule is much lower than that of HOCl. Fluorescence-based detection systems have emerged as very important tools to monitor biomolecules in living cells and organisms owing to distinct advantages, particularly the temporal and spatial sampling for in vivo imaging applications. To date, the development of HOBr-specific fluorescent probes is still proceeding quite slowly, and the research related to this area has not been systematically summarized. In this review, we are the first to review the progress made so far in fluorescent probes for selective recognition and detection of HOBr. The molecular structures, sensing mechanisms, and their successful applications of these probes as bioimaging agents are discussed here in detail. Importantly, we hope this review will call for more attention to this rising field, and that this could stimulate new future achievements.

## 1. Introduction

Nature contains a complex collection of elements, molecules, and ions that play irreplaceable roles in a wide range of chemical and biological processes. Selective recognition of these specific guests is a significant research area in supramolecular chemistry [1,2]. Among the various guests, reactive oxygen species (ROS), which have higher reactivity than molecular oxygen in the ground state, as their name suggests, are groups of reactive neutral and anionic small molecules [3]. In particular, intracellular ROS are produced within many cell types upon incomplete reduction of oxygen through multiple electron transfer reactions, depending on the cell and tissue types [4].

**Figure 1:**
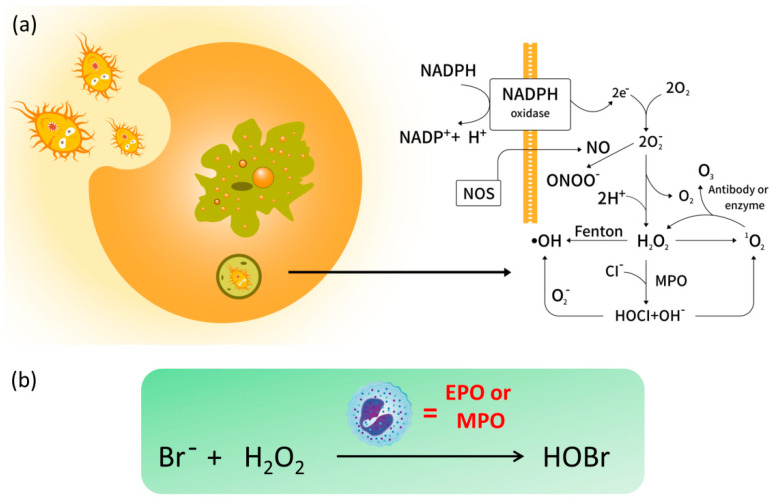
(**a**) The intracellular generation of reactive oxygen species (ROS) and (**b**) the formation of endogenous hypobromous acid (HOBr) catalyzed by eosinophil peroxidase (EPO) or myeloperoxidase (MPO).

One of the major sources of intracellular ROS is NADPH oxidase process (Figure 1a), and the commonly seen ROS include hydrogen peroxide (H_2_O_2_), hypohalous acids (HOCl/ClO^−^), superoxide anion (O_2_•^−^), hydroxyl radical (•OH), singlet oxygen (^1^O_2_), peroxynitrite (ONOO^−^) and ozone (O_3_) [5]. It has been widely demonstrated that endogenous ROS are involved in numerous biological signaling pathways [6,7,8]. However, excess production of ROS can lead to oxidative damage to a wide range of biomolecules such as carbohydrates, proteins, nucleic acids, and lipids, which has been implicated in physiological and pathophysiological processes such as cancer, aging, cardiovascular disease, diabetes mellitus, gastrointestinal diseases, Alzheimer’s disease (AD), and so on [9,10,11]. ROS concentrations within an expected threshold facilitate the dynamic balance of intracellular redox state. Undoubtedly, selective recognition and detection of those species have attracted a great deal of attention [12,13,14]. Not surprisingly, biologists and chemists have made joint efforts to conceive feasible ROS-related therapeutic approaches in the past decades. Nevertheless, the monitoring and quantitation of ROS remains a challenging task not only due to their high reactivity and short lifetime, but also to the low concentrations in vivo.

Hypobromous acid (HOBr) is an important ROS with a high similarity to HOCl in both chemical and physical properties. In organelles, HOBr is generated from the peroxidation of bromide anions (Br^−^) with H_2_O_2_ (Figure 1b), whose reaction is catalyzed by the heme peroxidases such as eosinophil peroxidase (EPO) or myeloperoxidase (MPO) [15]. Importantly, HOBr is a potent oxidizer with effective antibacterial activity, and is also regarded as an integral factor in the neutrophil host defense system. For instance, HOBr participates in the formation of sulfilimine cross-links in collagen IV, whose scaffolds are essential to the formation and function of basement membranes (BMs) in vivo [16]. In a similar fashion to other ROS, excessive generation of HOBr will injure organisms, which always results in inflammatory tissue damage and a variety of diseases [17,18,19]. Increasing evidence reveals that the EPO levels in serum of asthmatic patients is three times higher than that in healthy individuals [20]. Considering that the bromide concentration in blood and plasma is far lower than that of chloride (ca. 1000-fold) [21], the concentration of endogenous HOBr is relatively lower than HOCl, which renders the selective recognition of endogenous HOBr more challenging than other ROS.

Among various analytical techniques, fluorescent probes have emerged as indispensable tools to monitor biomolecules in living cells and organisms owing to the high sensitivity, non-invasive imaging, real-time detection, low cost, and superb spatio-temporal resolution [22,23,24,25]. Particularly, the probes with near-infrared (NIR) emission (650–900 nm) are highly favored for in bio-imaging due to their distinct ability of tissue penetration [26,27,28]. Meanwhile, ratiometric fluorescent probes are highly desirable because these molecules could overcome the ambiguities of single fluorescence intensity and offer quantitative measurements via self-calibration of two emission bands [29]. In addition, in contrast to aggregation-caused quenching (ACQ) that is quite common in most conjugated fluorescent molecular systems, the aggregation-induced emission (AIE) first discovered by Tang’s group in 2001 has become a major player in chemosensors [30,31,32,33,34].

To date, various classic fluorescence dyes, acting as signal reporters such as coumarins [35], 1,8-naphthalimides [36,37], rhodamines [38], difluoroboron dipyrromethenes (better known as BODIPY) [39,40,41,42], cyanine dyes [43], pyrene [44,45,46], AIE-active luminogens [47,48,49] and so on [50,51,52], have been widely developed to construct fluorescent chemosensors for broad and exciting applications. Recently, we have summarized small-molecule-based fluorescent probes for f-block metal ions [53] and pillararene-based receptors for binding of different metal ions [54] as well as fluorescent chemosensors and smart materials constructed from macrocyclic arenes that incorporate BODIPY [55]. Specifically, the reaction-based fluorescent probes (also known as chemodosimeters), whose recognition events involve irreversible chemical reactions as induced by a target analyte, have received great attention during the last decade as this promising and attractive strategy always offers high selectivity and sensitivity [56,57,58,59].

**Figure 2:**
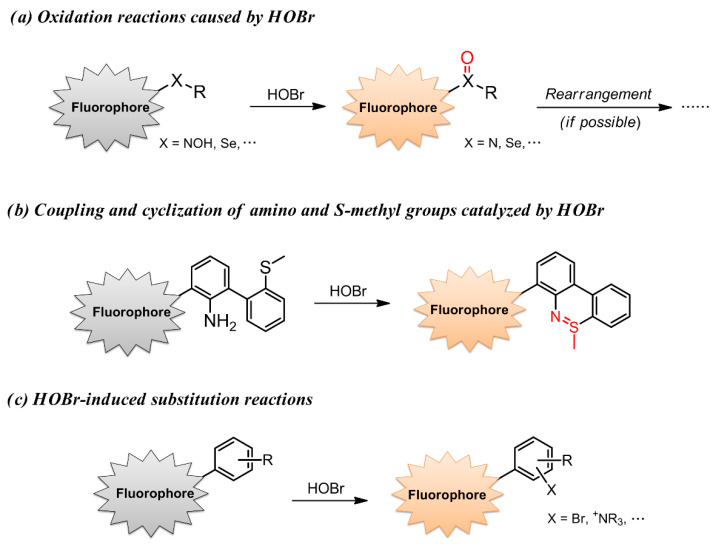
Three reported strategies to construct a suitable fluorescent probe for HOBr.

With regard to ROS, selective recognition of these reactive species using fluorescent detectors has been well documented in several excellent reviews, which mainly focus on •OH [60], HOCl/ClO^−^ [61], H_2_O_2_ [62], ONOO^−^ [63] and ^1^O_2_ [64]. Hitherto, the research related to fluorescent probes for selective recognition of HOBr has not been systematically summarized. Despite the growing interest, the development of HOBr-specific fluorescent probes is still evolving very slowly. Obviously, all the reported fluorescent probes for ROS function in a reaction-based manner [65,66,67], and HOBr is not an exception. There are three strategies to construct a suitable fluorescent probe for HOBr (Figure 2): (a) oxidation reactions caused by HOBr, where HOBr acts as a strong oxidant; (b) coupling cyclization of an amino group and S-methyl moiety catalyzed by HOBr; (c) HOBr-induced substitution reactions. In light of the recent achievements and deficiency in HOBr-specific fluorescence probes, this is a timely review of the advances made so far in constructions and applications of fluorescent probes for HOBr using diverse fluorophores.

In this mini review, we will provide all the literature of fluorescent probes for selective recognition and detection of HOBr, which represents the first comprehensive summary related to this attractive research area. The main text will be organized based on the three HOBr-sensing strategies as described above. The molecular structures, sensing mechanisms, and their successful applications as bioimaging agents will be discussed in detail. The purpose of this review is not only to provide a general overview of the design and development of fluorescent probes for HOBr, but more importantly, it calls for more attention to this rising field.

## 2. Probes Based on the Oxidation Reactions Caused by HOBr

**Figure 3:**
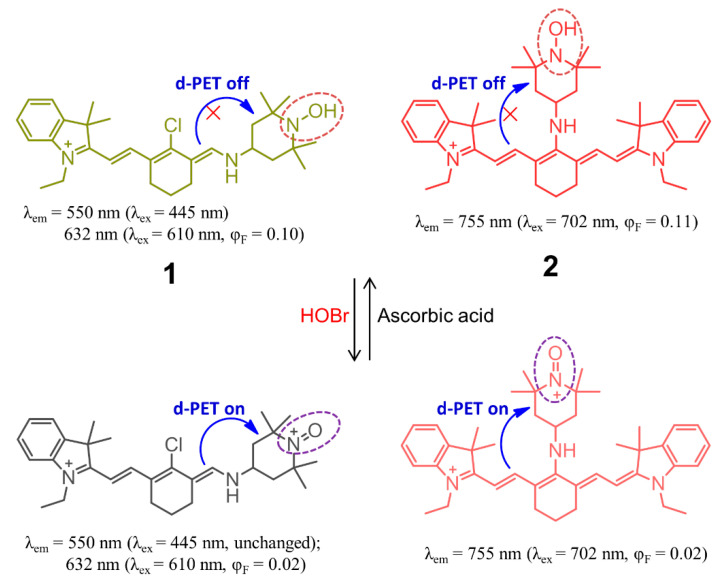
Chemical structures of cyanine-based fluorescent probes **1** and **2** for selective recognition of HOBr.

Not until 2012 did Han’s group report the construction of cyanine-based fluorescent probes **1** and **2** for the selective recognition of HOBr (Figure 3), which is the first example of a fluorescent probe for HOBr [68]. In this system, the cyanine platform served as a signaling fluorophore, while the 4-hydroxylamino-2,2,6,6-tetramethylpiperidine-N-oxyl (TemOH) moiety was incorporated both as the reaction site to HOBr and an effective fluorescent modulator. Despite an almost complete lack of changes in the absorption spectrum for the two probes upon treatment with HOBr under simulated physiological conditions (0.2 M PBS, pH = 7.4, 10 μM probes), a remarkable ratiometric fluorescence response for **1** and fluorescence quenching behavior for **2** were observed. The probe **1** exhibited two absorption maxima at 445 nm and 610 nm, together with the corresponding fluorescence maxima at 550 nm and 632 nm. The addition of HOBr did not cause a change of the fluorescence intensity at 550 nm but led to a decrease of the fluorescence intensity at 630 nm (quantum yield varying from 0.10 to 0.02), from which a 13-fold-decrease of the ratiometric fluorescence response F_632 nm_/F_550 nm_ could be deduced while increasing the amount of HOBr from 0 to 110 μM. This unique recognition mechanism could be ascribed to HOBr-mediated oxidation of the TemOH moiety to the corresponding oxyammonium cation, which resulted in a donor-excited PET (d-PET) quenching effect. In addition, further addition of ascorbic acid to the in situ system gave rise to the recovery of its fluorescence emission due to the ascorbic acid-induced reduction of the oxyammonium cation, resulting from the inhibition of the d-PET process. The fluorescence intensity could also be turned off and on repeatedly with the alternate addition of HOBr and ascorbic acid in at least three cycles. With regard to cyanine probe **2**, it exhibited NIR absorption and emission located at 702 nm and 755 nm (Φ = 0.11) in the same media, respectively. Similarly to **1**, compound **2** responded to the HOBr/ascorbic acid redox cycle in a sequential manner, but the latter dye suffered from serious bleaching after three cycles. Importantly, the two probes showed low toxicity to the RAW264.7 cell line and localized in the cytoplasm, and so they could be successfully used to monitor intracellular HOBr.

**Figure 4:**
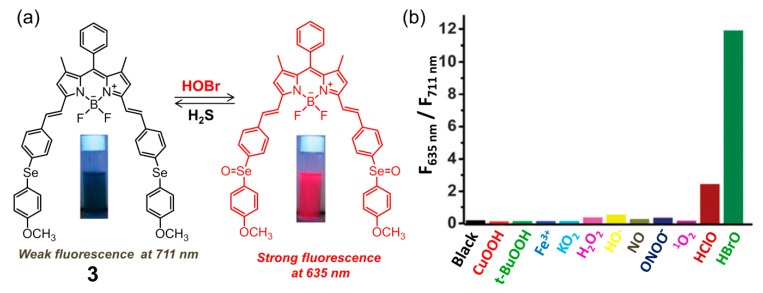
(**a**) Ratiometric fluorescent probe **3** on Se-BODIPY for selective recognition of HOBr followed by H_2_S in a redox cycle; (**b**) fluorescence intensity ratio (F_635 nm_/F_711 nm_) of **3** upon reacting with various ROS for 60 min. Reproduced with permission from Reference [69]. Copyright 2013 Royal Society of Chemistry.

Subsequently, the same group developed BODIPY-based fluorescent probe **3** for HOBr in H_2_O-CH_3_CN (4:1, *v*/*v*, 20 mM PBS, pH = 7.4) solution [69]. Structurally, probe **3** contained the 4-methoxyphenylselenide unit as a modulator (Figure 4a), which could not only extend the π-conjugation system of the fluorophore but also facilitated the fluorescence of the probe to tune to the NIR region owing to the strong electron-donating affinity of the Se atom. As expected, compound **3** showed very weak fluorescence with the maximum emission wavelength located NIR region (λ_em_ = 711 nm, Φ = 0.00083) due to the heavy atom effect and efficient PET process from the diarylselenides to the BODIPY backbone. In the presence of HOBr, the fluorescence emission was significantly blue-shifted to 635 nm (Φ = 0.206) with a 118-fold ratiometric (F_635 nm_/F_711 nm_) enhancement, which was also accompanied by an obvious color change from green to blue, indicating that probe **3** was an excellent colorimetric and ratiometric fluorescent sensor for HOBr. This recognition process could be attributed to the oxidation of selenide to selenoxide by HOBr, causing a shortening of the donor-acceptor π-conjugated system as the selenoxide has much higher electron-withdrawing ability. Actually, selenium-incorporating fluorescent probes have been widely demonstrated to be potent chemosensors for several ROS, including peroxynitrite and hypochlorous acid [70,71,72,73,74]. However, in this system, probe **3** was found to be highly selective towards recognition of HOBr and not liable to interference by other ROS (Figure 4b). Additionally, only the addition of H_2_S (with high selectivity over other reactive sulfur species such as Cys, Hcys and GSH) induced the reduction of selenoxide to the corresponding selenide, leading to recovery of its original fluorescence. The redox recognition event mediated by HOBr and H_2_S, whose detection limit is determined to be 50 nM and 0.1 μM respectively, could be repeated at least five cycles. The probe possessed good cell membrane permeability and was applied to continuously detect intracellular HOBr/H_2_S redox cycle replacement in RAW264.7 cells.

**Figure 5:**
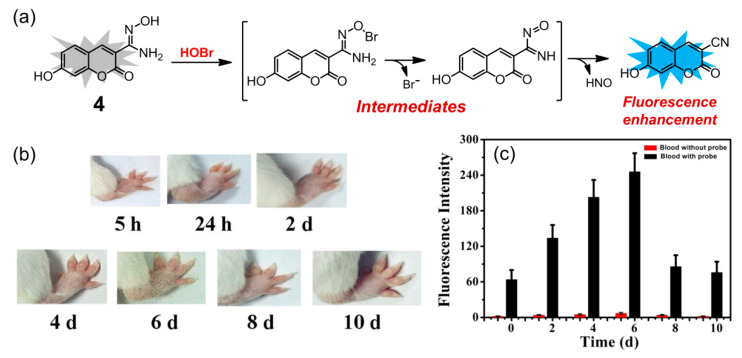
(**a**) Coumarin-based fluorescent probe **4** for HOBr; (**b**) swelling changes of right foot of mice within 10 days after injection of Freund’s adjuvant; (**c**) fluorescence response of probe **4** (50 μM) added in the blood of arthritis model mice within 10 days (λ_ex_ = 395 nm, slits: 5/5 nm). Reproduced with permission from Reference [75]. Copyright 2020 Elsevier.

In 2020, Zeng and coworkers reported a coumarin-based fluorescent probe **4** for selective detection of HOBr (Figure 5a), whose sensing mechanism was based on HOBr-mediated oxidation of amidoxime to the corresponding cyano group [75]. Probe **4** had good water solubility and exhibited a 5 nm red shift (from 395 to 400 nm) in its absorption spectrum in PBS buffer solution (pH = 7.4) upon addition of HOBr. On the other hand, the fluorescence intensity of **4** at 460 nm was greatly enhanced with the quantum yield varying from 0.0445 to 0.79 in the presence of HOBr, which could be ascribed to the ICT effect of the resultant product. The sensing ability was demonstrated to be optimal in the pH range of 7.0 to 8.0. This probe had a quite fast response time (less than 30 s) and the detection limit of HOBr was calculated as low as 30.6 nM. Probe **4** displayed a good selectivity for HOBr over other potential interferences, including ROS, RNS, various amino acids, and metal ions. Moreover, the content of HOBr in blood was monitored by the probe after successful establishment of an arthritic model mice (Figure 5b). The fluorescence intensity of **4** in the arthritic mice’s blood gradually increased within 6 days and reached a maximum value on the sixth day (Figure 5c), which was 4-fold higher than the value of untreated blood samples, indicating that probe **4** has potential for early diagnosis and evaluation of inflammation in clinical practice.

## 3. Coupling and Cyclization of Amino and S-Methyl Groups Catalyzed by HOBr

**Figure 6:**
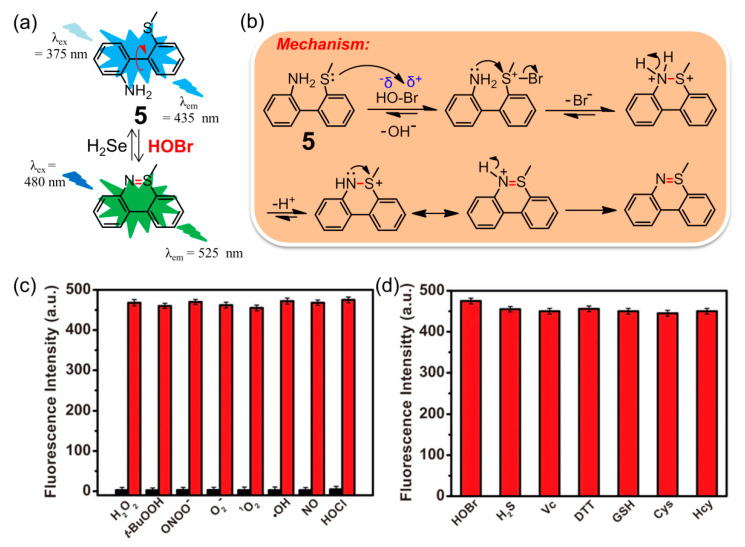
(**a**) Fluorescent probe **5** for selective recognition of HOBr followed H_2_Te; (**b**) the mechanism of oxidative cyclization of the amino group and S-methyl group catalyzed by HOBr; (**c**) fluorescence intensity of **5** after adding various active oxidizing species or (**d**) active reducing species. Reproduced with permission from Reference [76]. Copyright 2018 American Chemical Society.

Inspired by the key role that HOBr plays in the formation of sulfilimine (-S=N-) cross-links in collagen IV [16], Tang and coworkers pioneered the construction of a simple and ultrasensitive fluorescent probe **5** for selective recognition of HOBr (Figure 6a) [76]. This unique mechanism was based on a specific coupling cyclization between the amino group and S-methyl group catalyzed by HOBr (Figure 6b). The probe **5** was easily synthesized using commercially available o-(methylthio)-phenylboronic acid and o-bromoaniline through a Suzuki cross-coupling reaction in 85% yield. In PBS solution (pH = 7.4), the maximum excitation and emission wavelength of **5** located at 375 nm and 435 nm respectively, whereas those of the reaction product after treatment of **5** with HOBr were corresponding to 480 and 525 nm (Φ = 0.31). The large red shift of the maximum emission wavelength (ca. 90 nm) can be ascribed to the extended rigid skeleton of the reaction product. The probe featured an ultrasensitive response (detection limit of 17 pM), fast sensing time (approximate 3 min), low cytotoxicity (IC_50_ = 711.20 μM) and high selectivity for HOBr over other highly active oxidizing species (particularly the HOCl analogue, Figure 6c) and active reducing species (Figure 6d). Compared with probes **1–3** that detected HOBr only through activation after exposure to EPO, hydrogen peroxide (H_2_O_2_), and bromide anions (Br^−^) in live-cells, probes **5** imaged HOBr without bromine anion stimulation and exhibited different intensities of fluorescence emission with Br^−^, Br^−^/H_2_O_2_, or HOBr in HepG2 cells and zebrafish. Consequently, this probe ought to be a promising candidate for quantifying changes in endogenous HOBr, and is beneficial for a better understanding of the interconversion of Br^−^, Br^−^/H_2_O_2_, and HOBr in living organisms. The work shown not only presents the first example of a fluorescent probe for the specific detection of HOBr in vivo, but also it paves the way for acquiring other novel fluorescent sensors for HOBr with tailored properties (see the following examples). Soon afterwards, it was found that only H_2_Te reductant could trigger the recovery of the fluorescence to the original level of **5** as the sulfilimine bond can be easily cleaved by H_2_Te [77]. Thus, compound **5** was demonstrated to be an excellent sequential fluorescence sensor for HOBr followed by H_2_Te with high sensitivity and selectivity. The detection limit of H_2_Te was as low as 8.0 μM, and at least four cycles with a reasonable fluorescence decrement were acquired upon alternate addition of HOBr and H_2_Te. Furthermore, the cyclized product of **5** formed by HOBr could also be capable of detecting H_2_Te in HepG2 cells. Importantly, this HOBr/H_2_Te-mediated conjugated system worked very well in modulating the formation and cleavage of the sulfilimine bond in both dipeptide and C-terminal noncollagenous (NC1) hexamers, which offers a better understanding of the physiological function of collagen IV.

**Figure 7:**
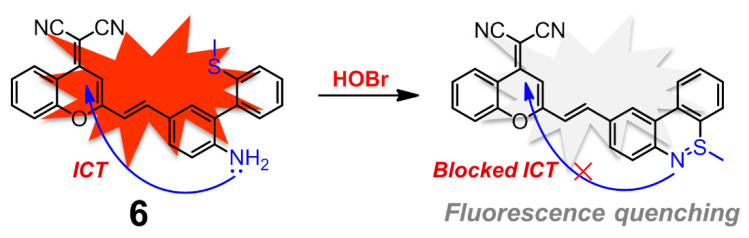
Dicyanomethylene-benzopyran (DCMB)-based near-infrared (NIR) fluorescent probe **6** for HOBr in a fluorescence quenching manner.

Dicyanomethylene-benzopyran (DCMB) derivatives are well-known NIR fluorophores with good stability and large Stokes shifts owing to the distinct push-pull effect and extended π-conjugation, which are ideal platforms to construct potent NIR fluorescent sensors for various analytes [78,79,80,81]. Conjugation between DCMB and the skeleton of compound **5** ought to afford a unique fluorescent probe for HOBr. As expected, compound **6** has demonstrated to be an excellent fluorescent probe for markedly selective recognition of HOBr over the ROS, reactive nitrogen species (RNS), reactive sulfur species (RSS), common biological amino acids and metal ions (Figure 7), although the probe worked in a fluorescence quenching manner [82]. The absorption centered at 478 nm of free probe **6** in PBS-CH_3_CN (3:2, *v*/*v*, pH 7.4) solution sharply decreased, accompanied by appearance of two new peaks at 392 nm and 448 nm in the presence of HOBr. Meanwhile, probe **6** showed strong fluorescence emission at 655 nm due to the efficient ICT process, which was mostly quenched and red-shifted to 700 nm together with a remarkable fluorescence color change from red to colorless after treatment with HOBr. The corresponding sensing mechanism could also be attributed to HOBr-triggered cyclization, and the reaction product lacked satisfactory ICT. The reaction kinetics of **6** with HOBr took about 8 min to level off. Probe **6** maintained its sensing ability in a wide pH range (pH = 4.0–8.0) with a detection limit of 0.66 μM. Moreover, this probe had a low cytotoxicity and was successfully applied for monitoring HOBr in MCF-7 cells.

**Figure 8:**
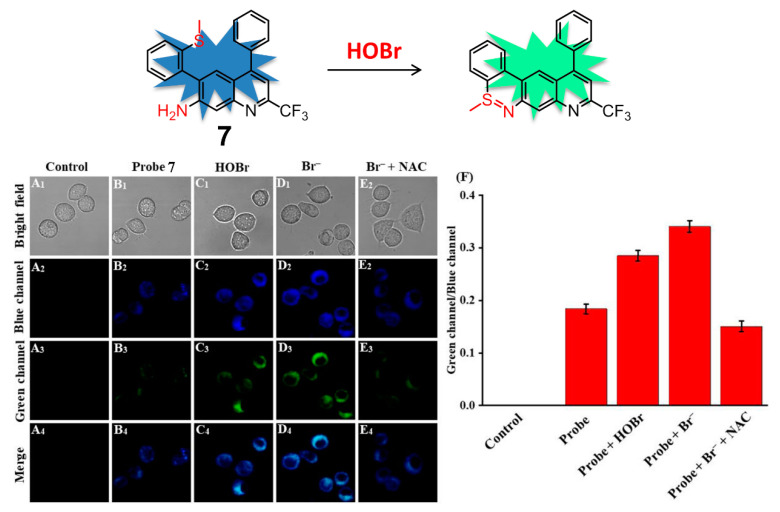
Ratiometric fluorescence probe **7** for HOBr (top) and its fluorescence imaging of HOBr in RAW 264.7 cells (down). Cells were incubated with (**A**) control; (**B**) probe **7**; (**C**) probe **7** then treated with HOBr (50 μM); (**D**) probe **7** then treated with NaBr (100 μM); (**E**) NaBr (100 μM) and NAC (100 μM, a scavenger of HOBr) then treated with probe **7**; (**F**) Fluorescence intensity ratios (F_green_/F_blue_) from (**A**–**E**). Blue channel: 455 nm–505 nm, green channel: 545 nm–645 nm, λ_ex_ = 434 nm. Reproduced with permission from Reference [83]. Copyright 2020 Elsevier.

Taking advantage of the same coupling cyclization of the amino group and S-methyl group, sulfilimine-based fluorescent probe **7** containing 4-CF_3_-7-aminoquinoline as the fluorophore for sensing HOBr was described in detail by Zhu and coworkers (Figure 8) [83]. Compound **7** displayed three intense absorption bands peaking at 275 nm, 335 nm, and 380 nm in PBS-EtOH (6:4, *v*/*v*, pH = 7.4) solution. The addition of HOBr induced a decrease of the absorption at 275 nm and 380 nm, as well as the emergence of two new absorption peaks appearing at 300 nm and 410 nm. The strong emission peak of **7** at 505 nm was gradually decreasing and a new emission signal started to appear at 545 nm along with a sustained increase of HOBr. The ratios of fluorescence intensity (F_545_/F_505_) offered a good linear correlation with the amount of HOBr in the range of 0–20 μM, from which the detection limit was estimated to be 92 nM. In addition, the low cytotoxicity and good biocompatibility of **7** also allowed the probe to recognize and image exogenous/endogenous HOBr in living RAW 264.7 cells and zebrafish.

Each of the distinct subcellular organelles plays an indispensable role in cellular processes, which requires an appropriate microenvironment and specific biological species to maintain their cellular functions [84,85]. Mitochondria, essential organelles in most eukaryotic organisms and the major contributor to cellular ROS levels, are responsible for energy supply and aerobic metabolism [86]. Consequently, monitoring mitochondrial microenvironments including ROS, pH, polarity, viscosity, and temperature may give more information on the status of this organelle [87,88,89,90].

**Figure 9:**
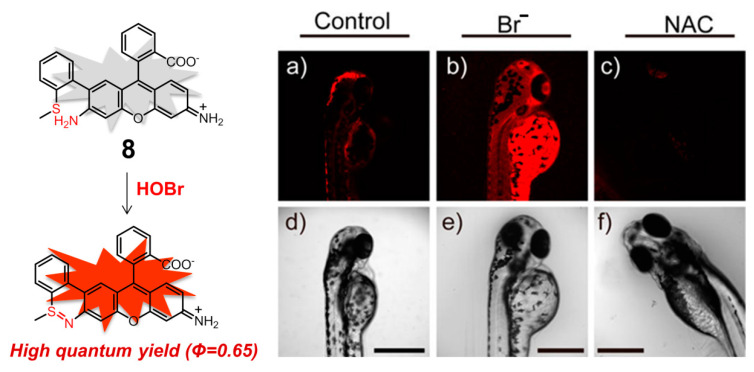
Mitochondria-targeting NIR fluorescent probe **8** for HOBr (left) and its fluorescence imaging of native HOBr in zebrafish (right). (**a**) Zebrafish fed with **8** for 30 min (50.0 μM); (**b**) zebrafish fed with Br^−^ (100 μM) for 30 min followed by **8** (50.0 μM) for another 30 min; (**c**) zebrafish incubated with NAC (a scavenger of HOBr,20.0 μM) for 30 min followed by **8** (50.0 μM) for another 30 min. (**d**–**f**) Correspond to bright-field images of (**a**–**c**). (λ_ex_ = 633 nm, λ_em_ = 650–750 nm). Reproduced with permission from Reference [91]. Copyright 2017 American Chemical Society.

On the basis of the same coupling/cyclization recognition mechanism, rhodamine derivative **8** (Figure 9) was rationally constructed by Tang and coworkers, which is the first example of a mitochondria-targeting fluorescent probe designed for monitoring native HOBr in vivo [91]. Rhodamine 110 can serve as both the fluorophore and the mitochondria-targeting unit, avoiding chemical modification of the parent dye with additional mitochondria-targeting groups. In HEPES buffer solution (10 mM, pH 7.4, containing 0.3% DMSO as a cosolvent), the probe **8** displayed the excitation and emission peaking at 495 and 530 nm, respectively, whereas the reaction product of **8** after treatment with HOBr showed the excitation and emission maxima in the NIR region, located at 624 and 663 nm, respectively. Benefitting from the high fluorescence quantum yield of the reaction product (Φ = 0.68) in the NIR region, which can greatly enlarge the signal-to-noise ratio and improves the detection sensitivity, probe **8** had an extremely low detection limit for HOBr (20 pM) and fast response time (ca. 3 min). Meanwhile, this molecule possessed low cytotoxicity (IC_50_ = 650 μM) and persistence of the fluorescence sensing ability for HOBr in a wide pH range (pH = 2.0–12). This probe did not exhibit any obvious interference by various bioanalytes including competing ROS, RNS, and commonly-seen metal ions and amino acids. It was also successfully utilized to image native HOBr in mitochondria of HepG2 cells and zebrafish. Taking the imaging of zebrafish as an example, in comparison to Figure 9a,c, a significant fluorescence enhancement was observed with the feeding of Br^−^ followed by probe **8** (Figure 9b), demonstrating that **8** was capable of monitoring the exogenous HOBr in vivo as HOBr can be only generated from Br^−^ in this case.

**Figure 10:**
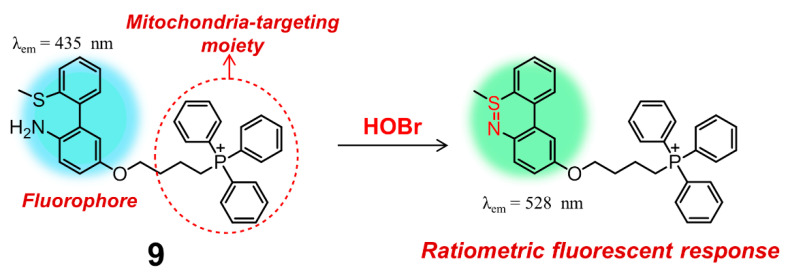
A ratiometric fluorescent probe **9** for bioimaging and biosensing of HOBr in mitochondria.

Apart from rhodamine dyes, these fluorophores decorated with one positively charged group, e.g., the classic pyridinium cation and triphenylphosphonium (TPP), can also have excellent mitochondrial targeting ability owing to the inherent charge attraction from the negative potential of inner mitochondrial membrane [92]. According to this strategy, Tian and Huang introduced a TPP group onto the backbone of **5**, leading to obtain another mitochondria-targeting ratiometric fluorescent probe **9** for selective recognition and biosensing of HOBr with high selectivity and sensitivity (Figure 10) [93]. In PBS solution (pH = 7.4, containing 0.5% DMSO), probe **9** behaved almost in the same fashion to **5**, indicative of the nearly-identical fluorescence emission centered at 437 nm. The initial emission of **9** gradually decreased with the addition of HOBr, accompanied by the enhancement of a new emission peak at 528 nm. Compound **9** had a faster response time (30 s) and a higher detection limit (1.8 ± 0.2 nM) for HOBr as compared to those of **5**. The low cytotoxicity, good biocompatibility and appreciable tolerance to a wide pH range (pH = 4.0–9.0) allowed probe **9** to function very well in real-time imaging and biosensing of HOBr in the mitochondria of RAW264.7 cells. More importantly, the work shown here clearly demonstrated that endogenous HOBr could also be generated from O_2_^•−^-induced oxidative stress rather than only the Br^−^ stimulation in mitochondria.

The lysosome is the main digestive compartment in the eukaryotic cell, where numerous macromolecules are degraded for cellular recycling [94]. Lysosome-targeting fluorescent probes have also been widely constructed for various biological species [95,96,97]. In particular, lysosome contains hydrolytic enzymes with a high proton concentration (pH < 6.0), meaning that it performs its function only under acidic conditions. Structurally, lysosome-localized fluorescent probes always incorporate lipophilic amines (e.g., morpholine and tertiary amine) [98]. These moieties can be easily protonated, which facilitate the positively charged probes to be entrapped and diffuse in the lysosomes.

**Figure 11:**
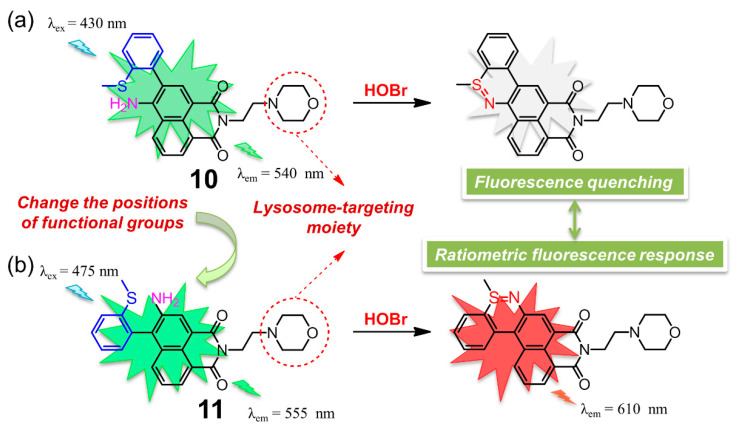
Chemical structures of two lysosome-targeting fluorescent probes (**a**) **10** and (**b**) **11** based on 1,8-naphthalimide framework for selective detection of HOBr.

Almost at the same time, two 1,8-naphthalimide-based fluorescent probes **10** [99] and **11** [100] for selective detection of HOBr in lysosomes were described in detail by two different groups (Figure 11). The two probes, containing the same morpholine group specifically designed for lysosome location, possessed a different position of the functional groups of 2-methylthiophenyl and amino groups. This slight structural variation between the two compounds did cause a distinctly different response towards HOBr in their photophysical or sensing properties. Naphthalimide derivative **10** was a two-photon fluorescent (88.8 GM) probe with a fluorescence quantum yield of 0.59 that functioned through a fluorescence switch-off manner. In HEPES solution (pH = 7.4, containing 0.1% DMSO), probe **10** displayed a strong absorption peak at 437 nm and a strong fluorescence centered at 540 nm, which was red-shifted to 451 nm and obviously quenched upon addition of HOBr. This on-off fluorescence phenomenon was attributed to efficient PET process of the reaction product, which was also confirmed by density functional theory (DFT) calculation. With respect to **11** in PBS buffer-CH_3_CN solution (3:2, *v*/*v*, 10 mM, pH = 7.4), the addition of HOBr led to a decrease of the emission of the free probe at 555 nm and the appearance of a new emission peak at 610 nm, accompanied by a prominent color transformation of the solution from yellow to red under 365 nm UV irradiation. The detection limits of **10** and **11** for HOBr were determined to be 33.5 nM and 99 nM, respectively. Both of the two probes showed good lysosome-targeting affinity, low cytotoxicity, fast response time (nearly seconds), high degree of selectivity and excellent persistence of sensing ability for HOBr in a wider pH range, which allowed it to work very well in imaging of HOBr in HeLa cells. Moreover, probe **10** was capable of detecting endogenous HOBr in living mice due to the nature of its two-photon properties. Importantly, the distinct recognition behavior retrieved from the slight structural difference in the present two examples can be useful for the design of other new fluorescent chemosensors with novel sensing performance.

## 4. Probes Based on Substitution Reactions Caused by HOBr

**Figure 12:**
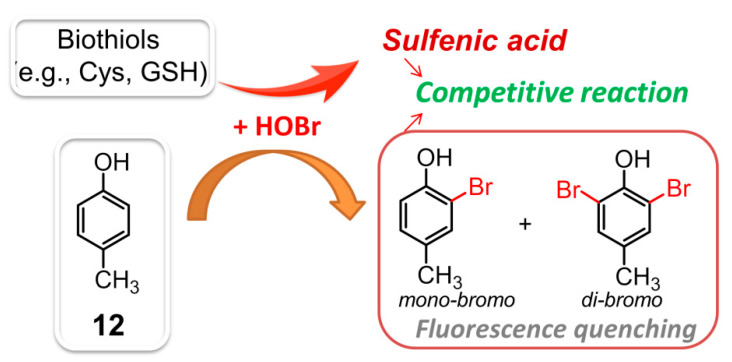
The competitive reaction of HOBr between **12** and biothiols.

Selective recognition and monitoring of HOBr can be achieved through substitution reactions, where HOBr acts as either the reactant or catalyst. In 2018, the first substitution-based fluorescent probe 4-methylphenol (*p*-cresol, **12**, λ_ex_ = 260 nm, λ_em_ = 305 nm) was described to react with HOBr (Figure 12), resulting in formation of nonfluorescent bromination products (i.e., monobromo-, dibromo-cresols) [101]. Under the specific conditions, compound **12** demonstrated a detection limit for HOBr down to 0.37 μM. Intriguingly, the biothiols (e.g., Cys, GSH, Hcy, etc.) could also react wih HOBr, giving rise to the corresponding oxidation product, sulfenic acid. The decreasing fluorescence intensity of **12** caused by this HOBr scavenging antioxidant was not comparable to that of the HOBr by itself. As a result, the competitive reaction of HOBr between **12** and biothiols also allowed the probe to determine the HOBr scavenging activity of various biothiols.

**Figure 13:**
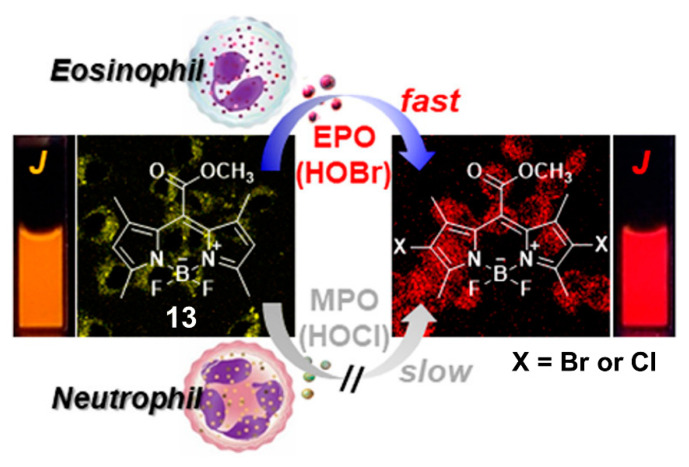
BODIPY-based fluorescent probe **13** for selective recognition of HOBr through dibrominated substitution. Reproduced with permission from Reference [103]. Copyright 2018 American Chemical Society.

Given the aggregation-induced emission enhancement (AIEE) properties of BODIPY derivative **13** through J-aggregation [102], Kim and coworkers demonstrated that **13** was also an efficient fluorescent probe for selective recognition of HOBr via dibromo substitution (Figure 13) [103]. In aqueous solution (100 mM acetate buffer, pH = 5.0, containing 0.1% CH_3_CN), this probe had the absorption and emission maxima centered at 580 and 581 nm, respectively. The addition of HOBr triggered the characteristic absorption and emission bands to decrease markedly and red-shift to 613 and 616 nm (ca. 22-fold fluorescence enhancement at 616 nm), respectively. These spectral changes could be ascribed to the suspended aggregates of the formed dibrominated product, which was confirmed by HPLC-MS analysis. HOBr also induced a naked-eye observable color change of the solution from pink to purple, together with a fluorescence color change from orange to red under UV lamp irradiation (365 nm). This probe showed extremely fast response (<2 s) for HOBr, and maintained its sensing ability in a pH range from 4.0 to 9.0. The emission ratio (F_616 nm_/F_581 nm_) of **13** was linearly dependent on the concentration of HOBr in the range of 1.0–5.0 μM, from which the detection limit was deduced to be 3.8 nM. The other ROS, and commonly-seen amino acids, biothiols as well as hydrolytic enzymes (including esterase, trypsin, lipase, lysozyme) did not interfere with the selective recognition of HOBr. Specifically for HOCl, this ROS could also react with **13** to give the dichlorinated product but even at a large excess amount ([HOCl]/[**13**] ≥ 80). As a result, probe **13** was halogenated with a much higher kinetic selectivity for HOBr over HOCl (≥1200 fold). Benefiting from the obtained advantageous photophysical properties, probe **13** was successfully applied to monitoring EPO activity and fluorescence assays of oxidative stress in cancer cells as well as immune response detection in live mice.

**Figure 14:**
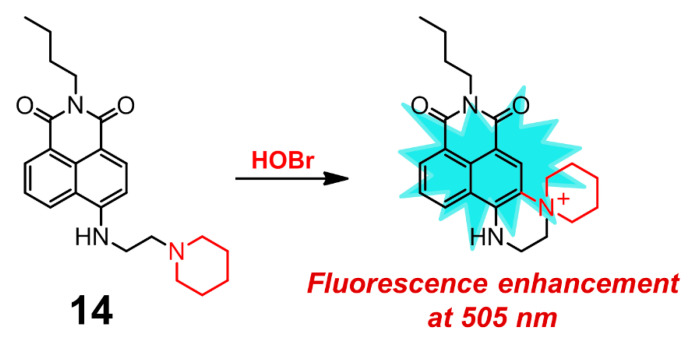
1,8-naphthalimide derivative **14** for selective detection of HOBr.

Zhu and coworkers presented 1,8-naphthalimide derivative **14** for the selective detection of HOBr (Figure 14), whose sensing mechanism was based on intramolecular substitution reaction mediated by HOBr rather than HOBr-acting as the brominating reactant [104]. Upon treatment with HOBr, the absorption band of **14** in H_2_O-EtOH (9:1, *v*/*v*, 20 mM PBS, pH = 7.4) shifted from 438 to 424 nm. Initially, probe **14** itself showed weak fluorescence at 530 nm, which was increased and blue-shifted to 505 nm in the presence of HOBr. Meanwhile, compound **14** exhibited a rapid response toward HOBr with the detection limit of 200 nM and reached its saturation point after about 1 min. This probe demonstrated no obvious interference from other ROS, RNS. and various biology-related metal ions. The obtained recognition behavior and good biocompatibility also allowed the probe to visualize HOBr both in RAW 264.7 cells and in zebrafish.

## 5. Summary and Outlook

The design and synthesis of fluorescent probes with specific recognition properties are quite fascinating as these molecules are amenable to biological imaging applications owing to their remarkable advantages of in vivo bioimaging analysis and real-time visualization. Selective and sensitive recognition of endogenous HOBr are in urgent demand, which can make a better understanding of its roles in numerous physiological and pathological processes. In this review, the advances made so far in fluorescent probes for HOBr have been discussed, which is the first comprehensive summary related to this area. The recognition properties were outlined in Table 1, and particular attention was paid to the design strategies, structural diversity, sensing mechanisms, and their applications.

Compared to other ROS, HOBr received relatively little attention, and the research area of fluorescent probes for HOBr has gained slow development since the first case was reported in 2012. Developing HOBr-specific fluorescent probes is an inter-disciplinary effort that requires the combined knowledge of organic chemistry, chemical biology and medicinal chemistry. Collectively, there are extensive challenges and opportunities in this emerging research field. In general, construction of new fluorescent probes containing versatile backbones with distinct recognition properties (e.g., high quantum yield, high photostability, rapid response time, low detection limit, high sensitivity and selectivity, low cytotoxicity, organelle-specificity, etc.) is significant, which will undoubtedly enrich the rare examples of fluorescent probes for HOBr. Specifically, the potential interference from HOCl ought to be taken into account when designing a fluorescent probe for HOBr with novel performance, since this reactive oxygen species has a high similarity to HOBr in both chemical and physical properties. Considering that there is only one reported example of a two-photon excitation probe and no case of second near-infrared biochannel (NIR-II, 1000–1700 nm) fluorescent probes for HOBr, more efforts should be made to conceive and study these luminescence probes. This is because these two types of fluorescent probes are crucial for long-time tracking of tissue, body-imaging, and biological processes due to their high deep penetration and low autofluorescence as well as time-resolved fluorescence imaging. A wide range of other types of probes for HOBr including fluorescent nanoparticles, nanoclusters, quantum dots, polymers, proteins, metal complexes (e.g., platinum, ruthenium, iridium, and rare earth complexes, MOF) have not been achieved, which ought to be the subject of initial studies. In addition, a sensing strategy for HOBr using supramolecular assembly has not been addressed.

Needless to say, the research of fluorescent probes for HOBr is just starting and still in its infancy, which leaves a great number of possibilities and opportunities. The review shown here not only provides a comprehensive summation of the construction and applications of fluorescent probes for HOBr, but we hope it will also be helpful for boosting this frontier field.

## Figures and Tables

**Table 1:** Summary of the fluorescent probes for HOBr.

Entry	Probe	Solvent System	Signal Type	λ_ex/_λ_em_ (nm)	Response Time	Applications	Detection Limit	Ref.
1	**1**	0.2 M PBS (pH = 7.4)	ratiometric	445/550; 610/632	900 s	HOBr-imaging in RAW264.7 cells	n.d. *^a^*	[68]
2	**2**	0.2 M PBS (pH = 7.4)	turn-off	702/755	900 s	HOBr-imaging in RAW264.7 cells	n.d. *^a^*	[68]
3	**3**	20 mM PBS containing 20% CH_3_CN (pH = 7.4)	ratiometric	610/635, 711	3.0 min	HOBr-imaging in RAW264.7 cells	50 nM	[69]
4	**4**	10 mM PBS (pH = 7.4)	turn-on	395/460	30 s	monitoring HOBr in arthritis model mice and real-time evaluating the development of arthritis	30.6 nM	[75]
5	**5**	10 mM PBS containing 0.5% CH_3_CN (pH = 7.4)	turn-on	480/525	ca. 3.0 min	imaging endogenous HOBr in HepG2 cells and zebrafish	17 pM	[76]
6	**6**	10 mM PBS -CH_3_CN (3: 2, *v*/*v*, pH = 7.4)	turn-off	488/655	8.0 min	monitoring HOBr in MCF-7 cells	660 nM	[82]
7	**7**	10 mM PBS-EtOH (6:4, *v*/*v*, pH = 7.4).	ratiometric	460/505, 545	50 s	tracking the changes of HOBr in RAW 264.7 cells and zebrafish	92 nM	[83]
8	**8**	10 mM HEPES containing 0.3% DMSO (pH = 7.4)	turn-on	624/663	ca. 3.0 min	imaging native HOBr in mitochondria of HepG2 cells and zebrafish	20 pM	[91]
9	**9**	10 mM PBS containing 0.5% DMSO (pH = 7.4)	ratiometric	405/437, 528	30 s	imaging of HOBr in mitochondria of RAW264.7 cells	18 nM	[93]
10	**10**	10 mM HEPES containing 0.1% DMSO (pH = 7.4)	turn-off	430/540	immediately	imaging of exogenous and endogenous HOBr in Hela cells and mice	33.5 nM	[99]
11	**11**	10mM PBS-CH_3_CN (3:2, *v*/*v*, pH = 7.4)	ratiometric	475/555, 610	12 s	imaging of exogenous and endogenous HOBr in HeLa cells	99 nM	[100]
12	**12**	distilled water	turn-off	260/305	n.d. *^a^*	determination of the HOBr scavenging activity of biothiols and some pharmaceutical samples	0.37 μM	[101]
13	**13**	100 mM acetate buffer containing 0.1% CH_3_CN (pH = 5.0)	ratiometric	480/581, 616	≤2 s	monitoring EPO activity and fluorescence assays of oxidative stress in cancer cells (HCT116 and A549) as well as immune response detection in live mice.	3.8 nM	[103]

*^a^* n.d. means not determined.

## Data Availability

Not applicable.
